# Informal and formal workforce training and dementia care competencies across long-term care settings in the U.S.

**DOI:** 10.3389/frhs.2026.1894813

**Published:** 2026-08-05

**Authors:** Sunshine M. Rote, Heehyul Moon, Kathryn Williams Sites

**Affiliations:** Kent School of Social Work and Family Science, University of Louisville, Louisville, KY, United States

**Keywords:** assisted living communities, dementia, knowledge - attitude - behavior, nursing home (NH), training, workforce & research

## Abstract

**Introduction:**

This study uses data on the dementia care workforce in the U.S. to examine whether the relationship between formal and informal training and dementia care knowledge, attitudes, and practices vary across long-term care settings.

**Methods:**

Data is drawn from the National Dementia Workforce Study (NDWS, wave 1 2024–2025). Logistic regression analyses are used to assess the relationships between training and dementia care competencies and moderation effects by memory care unit for nursing home staff (*N* = 355) and assisted living staff (*N* = 404).

**Results:**

For nursing home staff, both formal and informal training are associated with greater perceived confidence in dementia care. For assisted living staff, only formal training is associated with more person-centered knowledge about persons living with dementia. Interaction effects of training type by frequency of care in a memory care unit demonstrate that informal training is more impactful for dementia knowledge for assisted living staff who never provide care in memory care units compared to those who provide care all the time in memory care units.

**Discussion:**

Findings demonstrate a need for a differentiated training investment strategy responsive to the care environment. In countries or care systems where dedicated memory care units are not common or underfunded, in addition to formal credentialing, informal training is a potentially important workforce development tool to improve both worker and patient outcomes.

## 1 Introduction

According to the World Health Organization (1), dementia is the seventh leading cause of death and one of the major causes of dependency for older adults globally. One of the proposed actions outlined in the WHO's global action plan on public health responses to dementia is for member states to earmark resources and budgets for building knowledge and skills of their dementia care workforces (2). Adequately trained and qualified workforces, informed by evidence-based and person-centered care, are required in both informal settings (i.e., homes and communities) and formal settings (i.e., nursing homes and assisted living facilities) to ensure quality of care. However, the World Health Organization (2) notes that a significant portion of healthcare and dementia care workforces globally do not receive adequate and ongoing training, supervision, and support to aid in career development and improve the quality of care provided (3).

This action may become increasingly pressing as projected number of available informal, unpaid caregivers globally is expected to shrink, placing increasing pressure on formal and long-term care workforces to meet this growing demand. For example, in the U.S. to maintain the current worker-to-older-resident ratio an additional 3.5 million long-term care healthcare workers will be needed by 2030 (4). Translating this global call into actionable and context-specific policy requires identifying existing training and knowledge gaps within current workforces Additionally, establishing training and knowledge gaps within the existing workforces is needed in order to identify mechanisms to improve the quality of dementia care being provided (5, 6). This study addresses a knowledge gap by examining training and knowledge deficits within the U.S. dementia care workforce specifically, where distinct regulatory and financing structures shape how such recommendations can be implemented.

Dementia service providers in residential care settings have expressed a desire for more practical learning methods and applications, as well as training that is relevant to their daily work, including real-life situations and solutions (7–9). Well-designed training infrastructures that strengthens staff knowledge, skills, and confidence may therefore simultaneously improve worker outcomes and residents' quality of care (10, 11). Meeting the training needs of long-term care staff may also improve worker retention, reduce workplace turnover, and foster career growth and confidence while reducing work-related stress (6, 12).

There is evidence that formal training or more structured instruction improves specialized dementia care knowledge and to a lesser extent attitudes about dementia (12, 13). Two instruments are particularly relevant to measuring these outcomes. The Dementia Attitudes Scale (DAS), developed by O'Connor and McFadden (14), assesses two dimensions: dementia knowledge (including beliefs about dementia, personhood, capacity) and dementia social comfort (the degree to which one is at ease, willing, and positively oriented towards interacting with persons living with dementia). Formal training has been shown to improve scores on the DAS (13). The Sense of Competence in Dementia Scale (SCIDS) focuses on how confident staff feel in their care-related abilities and practices (15). Training has been associated with gains in both worker competence and confidence (16). Together, both the DAS and the SCIDS offer complementary coverage of attitudinal and practice-oriented outcomes and are well-suited for examining how different training experiences shape workers' capabilities.

Despite existing evidence, the training in the dementia care workforce literature has important limitations. Most studies focus narrowly on formal training and dementia knowledge and tend to draw from convenience samples of high-performing facilities rather than a representative cross-section of the workforce. Informal, on-the-job, learning through peer mentoring, supervisor modeling, and accumulated experience may shape attitudes in distinct ways from formal instruction, yet few studies compare across informal and formal training. Moreover, prior studies have not examined whether training type differentially predicts attitudinal vs. practice-oriented outcomes, nor have they compared these relationships across care settings (nursing homes vs. assisted living facilities).

In the U.S., in particular, ffederal regulations require nursing homes and assisted living communities to maintain formal training programs for all new and existing staff in specific content areas including dementia care management and abuse prevention with certified nursing assistants required to complete a minimum of 12 h of in-service dementia-focused training (42 CFR 483.95). Many states with the U.S. have developed additional training requirements; however, these vary considerably in content, required hours, and the staff roles to which they apply (17). This regulatory variability, combined with the gap in comparative research, limits our understanding of how training functions across different long-term care contexts.

However, there is reason to expect that training may operate differently across care settings, given the meaningful differences in resident acuity, staffing patterns, and the care environment itself. Nursing homes (NHs) and assisted living (AL) facilities serve residents with varying levels of functional and cognitive need, and their staffing composition structures reflect those differences (18). For example, nursing homes serve residents with higher acuity needs and operate under federal staffing mandates, whereas assisted living facilities vary widely in size, ownership, and the proportion of residents living with dementia (18). Specialized memory care units, which provide a setting for more tailored to the cognitive and functional needs of residents, represent a further distinct context. These include modification of the environment in which care is provided, specialized practices, as well as additional training requirements in dementia care (19).

Previous research has not systematically compared the relationship between training type and dementia knowledge, attitudes, and care practices across NH and AL settings, including memory care units. The present study addresses this gap by using both the DAS and SCIDS and their respective subdomains to examine whether formal and informal training differentially predicts attitudinal and practice-oriented outcomes, and whether these relationships vary by care setting. The current secondary data analysis project uses national data from wave 1 of the National Dementia Workforce Study (NDWS, *N* = 759, 2024–2025), which allows for extrapolation to the larger dementia care workforce in the U.S (18, 20). Our specific research questions are:

**RQ1**: Is informal and formal training associated with dementia care knowledge, attitudes, and practices for staff in nursing homes and assisted living facilities?
**RQ2**. Does frequency of care provision in memory care units moderate the association between training type and dementia care knowledge, attitudes, and practices among long-term care staff?

## 2 Materials and methods

### 2.1 Data

The current study uses data from wave 1 of The National Dementia Workforce Study (NDWS, 2024–2025) the first national survey of dementia care workforce professionals in the U.S (20). The NDWS was funded by the National Institute on Aging (U54AG084520) and designed to address AD/ADRD Research Implementation Milestone 13.Y by systematically characterizing the dementia care workforce across settings (20). Organizations and staff were recruited using a stratified probability sampling design, with explicit stratification by facility size, rurality, and proportion of Medicaid/low-income residents to ensure national representativeness (18). Wave 1 data was collected between August 2024 and March 2025 across nursing homes and assisted living facilities with response rates of 67.4% for staff in assisted living facilities and 55.2% for staff in nursing homes (18), and there are plans to collect this data on an annual basis across care settings. For the current analysis, we include respondents (*N* = 759) who have complete data across our study variables. Given the different sampling strategies and approaches for the nursing home staff and assisted living staff samples, we estimate models separately for these two groups. Our final analytic sample included 355 nursing home respondents and 404 assisted living respondents, representing over 90% of the total sample in both the nursing home and assisted living settings.

### 2.2 Measures

#### 2.2.1 Dependent variables

The shortened version of **Dementia Attitudes Scale (DAS)** assesses dementia care knowledge and attitudes and includes three items from each of the comfort and person-centered knowledge subscales with all items on a 7-point Likert scale (7 = strongly agree; 1 = strongly disagree) (14). The comfort subscale items include: “it is rewarding to work with people who have dementia,” “I am comfortable touching people with dementia,” and “I feel relaxed around people with dementia.” The person-centered knowledge subscale include: “people with dementia can be creative,” “it is possible to enjoy interacting with people with dementia,” and “people with dementia can enjoy life” (21). Responses were summed across both the full scale and subscales. The DAS demonstrated good to excellent reliability in the analytic sample across settings. Cronbach's alpha was 0.89 for the full scale and 0.94 and 0.85 for both the comfort and person-centered subscales among assisted living respondents. For nursing home respondents, Cronbach's alpha for the full scale was 0.92 and 0.83 and 0.90 for the comfort and person-centered subscales respectively.

Responses to the DAS were highly skewed with over 20% of assisted living and nursing home respondents answering “strongly agree” for every item, precluding the treatment of this variable as continuous. Therefore, a binary variable was created with total scores of less than 35 coded as low and scores greater than or equal to 35 coded as high. For the comfort subscale, scores less than 17 were coded as low and scores greater than or equal to 17 were coded as high. For the person-centered subscale, scores less than 18 were coded as low and scores greater than or equal to 18 were coded as high. These cut points were determined so as not to divide the heavily clustered upper range of responses. The low and high categories approximate a split between the bottom 25% of respondents and top 75%, although specific split values varied for setting and subscales.

The shortened version of the **Sense of Competence in Dementia Care (SCIDS)** assesses the perceptions associated with practices using from the full scale in the subdomains of professionalism (5 items) and sustaining personhood (4 items) using a 4-point Likert scale (4 = very confident, 1 = not at all confident) (15). Professionalism subscale items includes “I can keep up a positive attitude towards residents with dementia,” “I can keep up a positive attitude towards the relative of residents with dementia,” “I can keep myself motivated during the working day,” “I can play an active role in my team,” and “I can deal with personal care, such as incontinence, in a resident with dementia.” Sustaining personhood subscale items include “I can use information about their past when talking to a client with dementia,” “I can change my work to match the changing needs of a client with dementia,” “I can protect the dignity of a client with dementia,” and “I can offer choice to a client with dementia.” The SCIDS demonstrated acceptability to good reliability with a Cronbach's alpha of 0.87 and 0.88 for the total scale among assisted living and nursing home respondents respectively. Cronbach's alpha for the professionalism subscale was 0.80 among assisted living respondents and 0.83 among nursing home respondents. For the personhood subscale, Cronbach's alpha was 0.76 for the assisted living respondents and 0.77 for the nursing home respondents.

Responses to the SCIDS were highly skewed with over 40% of assisted living and nursing home respondents reporting “very confident” for each item. Therefore, following the same procedure as outlined in the DAS, the SCIDS total scale and subscales were operationalized as binary variables. Total scores less than 34 were coded as low and scores greater than or equal to 34 were coded as high. For the professionalism subscale, scores less than 19 were coded as low and scores greater than or equal to 19 were coded as high. For the personhood subscale, responses less than 15 were coded as low and responses greater than or equal to 15 were coded as high. As with the DAS, these cut points were selected to avoid splitting the heavily cluster upper range of responses and the low and high categories approximate a split between the bottom 25% of respondents and top 75% of respondents, with specific split values varying for setting and subscales.

#### 2.2.2 Independent variables

Participants were asked “have you ever received **formal training** (online or in-person) in…” and provided the option to respond yes or no to training in 10 content areas which included: (1) understanding dementia, (2) direct care skills such as responding to clients' behavior, (3) communicating with people with dementia, (4) working with families of people with dementia, (5) identifying changes in clients' condition, (6) providing end of life care, (7) caring for clients of different culture, values, or beliefs, (8) respecting clients' rights, (9) protecting clients against injury, and (10) projecting yourself against injury. Participants were also asked “have you ever received **informal on-the-job training** or completed self-study in…” the same 10 content areas with response options of “yes” or “no” for each content area. Training completion was highly skewed with 86% of nursing home respondents receiving formal training in all ten areas and 87% receiving informal training in all ten areas. Among assisted living respondents, 81% received formal training in all ten areas and 79% received informal training in all ten areas. Therefore, formal and informal training was operationalized as a binary variable (i.e., all training vs. some training). Respondents who endorsed all training items were coded as having had “all training” while those who failed to endorse at least one item were coded as having “some training”.

Finally, respondents were asked how frequently they work in a **memory care unit** with response options all of the time, some of the time, and never (reference category).

#### 2.2.3 Control variables

Sociodemographic control variables included gender [female/women vs. male/man (reference category)], race and ethnicity [white only and non-Hispanic (reference category), Black only and non-Hispanic, Hispanic, and other/multiracial]), nativity status [born within a United States state or territory (reference category) or born outside the United States], age [born before 1965, born between 1965 and 1985 (reference category), and born after 1985], and education [high school or less (reference category) and more than high school].

Work related control variables included hours paid to work in this job per week, length at current job, licensure status, supervisory status, direct dementia care provision, and resident assignment consistency. Hours worked per week was recorded as a continuous variable from 1 to 125. Licensure was coded as a binary variable indicating whether respondents currently hold an active CNA, HHA, LPN, MA, or other professional license with no license as the reference category. Job length was coded as having over one year with current employer or less than one year with the current employer (reference category). Participants were considered supervisors if they answered affirmatively that they supervise others in their role with non-supervision as the reference category. Participants were asked if they provided direct care to people with dementia (yes/no), with not providing direct dementia care used as the reference category. Resident assignment category was measured using three categories: residents stay the same (reference category), residents change, or both.

### 2.3 Analysis

**For RQ1,** we will assess whether there are significant differences in dementia care knowledge, attitudes, and practices by informal and formal training in both nursing homes and assisted living facilities using logistic regression and controlling for demographic and work-related factors. In these models, we examined informal and formal training separately in order to assess the associations of each training type with our outcomes separately. **For RQ 2,** this analysis will focus on whether the impact of formal and informal training on dementia care knowledge, attitudes, and practices varies across how often staff provide care in a memory care unit. All analyses were performed in STATA18 and include sample weights and our significant threshold was *p* < .05

## 3 Results

Table 1 provides an overview of the study variables. A large portion of staff working in nursing homes (86%) and assisted living facilities (81%) have completed formal training across all content areas. Similar rates of informal training completion were also reported across settings. More nursing home and assisted living staff report care in memory care units none of the time (35% & 39%) than all of the time (22% and 29%). Most staff report direct dementia care provision in both settings. In terms of demographics, most identify as women, non-Hispanic White, and most were born within the U.S. About half of staff in both settings were born after 1985 and more nursing home staff had more than a high school education (66%) compared with assisted living staff (48%). While 86% of the nursing home staff had a license, about 43% of assisted living staff were licensed. Nursing home staff on average report 40 h of work per week while assisted living staff reported about 36 h per week. Most staff have been with their current employer for over a year in both settings, and less than half of the staff report that they work with the same residents every day.

**Table 1:** Descriptive statistics.

Study variables	Percentage or mean (SD)	
	Nursing home	Assisted living
	(*N* = 355)	(*N* = 404)
Formal Training		
All formal training	86.46%	81.13%
Some formal training	13.54%	18.87%
Informal Training		
All formal training	87.27%	79.03%
Some formal training	12.73%	20.97%
Memory Care Unit		
All of the time	22.39%	29.13%
Some of the time	38.64%	35.56%
None of the time	38.97%	35.31%
Direct Dementia Care		
Provides direct dementia care	93.25%	89.51%
Does not provide direct dementia care	6.75%	10.49%
Female	93.40%	86.17%
Race/ethnicity		
Non-Hispanic White	43.67%	59.56%
Black	35.35%	14.92%
Hispanic	13.61%	10.36%
Other/multiracial	7.36%	15.16%
Birth Year		
Before 1965	9.52%	13.13%
1965 to 1985	42.66%	31.22%
After 1985	47.82%	55.64%
Birthplace		
US State or Territory	94.20%	87.89%
Outside the US	5.80%	12.11%
Education		
Highschool or less	33.99%	52.02%
More than high school	66.01%	47.98%
License		
Has license	85.48%	42.60%
No license	14.52%	57.40%
Hours per week	39.76 (63.64)	36.49 (13.14)
Length at employer		
Less than one year	32.38%	19.36%
Over one year	67.62%	80.64%
Working with residents		
Residents stay the same	38.18%	64.08%
Residents change	12.74%	6.15%
Both	49.08%	29.77%
Supervisor	33.97%	37.12%

Sample weights were applied.

### 3.1 Training and the DAS

Table 2 presents the results from the overall DAS scale as well as comfort and person-centered knowledge subscales. For the nursing home staff data, informal training is not significantly related to the DAS total or the comfort and person-centered knowledge subscales, whereas formal training is significantly associated with the DAS person-centered knowledge subscale (OR = 2.68; 95% CI: 1.25–5.73) but not the DAS total or comfort subscale. There are significant differences across how often the staff provides care in a memory care unit; however, the results are surprising and unexpected in that staff who provide care all of the time in memory care units report lower scores on the DAS person-centered knowledge subscale relative to the caregivers who never provide care in a memory care unit (OR = 0.42 95% CI: 0.20–0.86). In results not shown, other significant predictors of higher scores on the DAS include younger age.

**Table 2:** Logistic regressions of dementia knowledge and attitudes by training and provision of care in memory care unit (NDWS).

Independent variables	DAS total		DAS comfort		DAS person-centered knowledge	
	Formal	Informal	Formal	Informal	Formal	Informal
	Nursing Home Staff (*N* = 355)					
Training	1.69 [0.61, 4.70]	1.17 [0.39, 3.57]	2.15 [0.79, 5.83]	2.14 [0.77, 5.96]	2.68 [1.25, 5.73]*	0.98 [0.34, 2.83]
Memory Care Unit vs. None of the Time						
All of the Time	0.76 [0.37, 1.59]	0.79 [0.40, 1.56]	0.58 [0.23, 1.46]	0.58 [0.24, 1.41]	0.42 [0.20, 0.86]*	0.46 [0.25, 0.85]*
Some of the Time	1.34 [0.66, 2.73]	1.40 [0.69, 2.86]	1.68 [0.69, 4.13]	1.79 [0.74, 4.30]	1.47 [0.45, 4.78]	1.59 [0.52, 4.84]
	Assisted Living Staff (*N* = 404)					
Training	5.61 [1.21, 26.03]*	2.32 [0.84, 6.38]	2.01 [0.55, 7.35]	1.01 [0.39, 2.63]	4.91 [2.03, 11.87]**	2.92 [0.99, 8.58]
Memory Care Unit vs. None of the Time						
All of the Time	1.99 [0.31, 12.53]	3.10 [0.56, 17.02]	5.78 [1.71, 19.57]**	7.31 [2.56, 20.97]*	0.75 [0.18, 3.20]	1.03 [0.20, 5.36]
Some of the Time	0.65 [0.22, 1.92]	0.83 [0.30, 2.65]	1.70 [0.59, 4.94]	1.94 [0.71, 5.31]	0.76 [0.28, 2.02]	0.92 [0.33, 2.57]

Odds Ratios with 95% CI in parentheses; weighted data; controlling for sociodemographic and work-related characteristics.

** *p* < .01.

* *p* < .05.

For the data from staff in assisted living facilities, in contrast, we find that formal training is associated with greater likelihood of scoring in the high group for both the DAS total (OR = 5.61 95% CI: 1.21–26.03) and the DAS person-centered subscale (OR = 4.91 95% CI: 2.03–11.87). While training was not significantly related to the DAS comfort subscale, frequency of care provided in memory care units was a significant predictor with staff who provide care all of the time having a greater likelihood of scoring in the high group of the DAS comfort subscale (OR = 5.78; 95% CI: 1.71–19.57).

### 3.2 Training and SCIDS

Table 3 focuses on SCIDs and professionalism and sustaining personhood subscales. Overall, we find that both informal and formal training are significantly related to SCIDS total (OR = 3.67; 95% CI: 1.75–7.69; OR = 3.80; 95% CI: 1.60–9.02), SCIDs professionalism subscale (OR = 3.79; 95% CI: 1.64–8.78; OR = 2.98; 95% CI: 1.67–5.31], and SCIDS sustaining personhood subscale (OR = 3.76; 95% CI: 1.69–8.33; OR = 5.82; 95% CI: 2.52–13.42) among nursing home staff. For staff in assisted living facilities, formal and informal training were not significantly related to the SCIDs or SCIDs subscales. Finally, frequency of care in memory care units was not significantly related to the SCIDS for either nursing home or assisted living staff.

**Table 3:** Logistic regressions of dementia care practices by training and provision of care in memory care unit (NDWS).

Independent variables	SCIDS total		SCIDS Professionalism		SCIDS sustaining personhood	
	Formal	Informal	Formal	Informal	Formal	Informal
	Nursing Home Staff (*N* = 355)					
Training	3.67 [1.75, 7.69]**	3.80 [1.60, 9.02]**	2.98 [1.67, 5.31]**	3.79 [1.64, 8.78]**	5.82 [2.52, 13.42]***	3.76 [1.69, 8.33]**
Memory Care Unit vs. None of the Time						
All of the Time	0.95 [0.25, 3.56]	0.95 [0.26, 3.48]	0.81 [0.20, 3.19]	0.79 [0.20, 3.14]	1.45 [0.70, 3.00]	1.45 [0.75, 2.82]
Some of the Time	1.73 [0.60, 4.95]	1.91 [0.76, 4.78]	2.02 [0.81, 5.03]	2.14 [0.95, 4.84]	1.91 [0.62, 5.91]	2.18 [0.78, 6.08]
	Assisted Living Staff (*N* = 404)					
Training	3.96 [0.69, 22.88]	1.27 [0.25, 6.52]	1.35 [0.19, 9.66]	0.58 [0.13, 2.52]	2.67 [0.49, 14.55]	1.21 [0.15, 9.55]
Memory Care Unit vs. None of the Time						
All of the Time	0.48 [0.12, 1.96]	0.66 [0.23, 1.88]	0.71 [0.13, 4.04]	0.85 [0.18, 3.90]	0.51 [0.12, 2.24]	0.63 [0.19, 2.11]
Some of the Time	0.55 [0.20, 1.50]	0.69 [0.33, 1.44]	2.07 [0.59, 7.35]	2.32 [0.64, 8.42]	0.55 [0.20, 1.52]	0.65 [0.23, 1.87]

Odds Ratios with 95% CI in parentheses; weighted data; controlling for sociodemographic and work-related characteristics.

*** *p* < .001.

** *p* < .01.

### 3.3 Moderation effects by memory care unit

In Table 4, we assessed whether there were significant interaction effects of training* frequency of care in memory care units for the total DAS and total SCIDS across both settings. The only significant interaction observed was for informal training*memory care unit for the DAS total and only in assisted living facilities. Supplementary Figure S1 depicts these significant interaction effects. Overall, we found little differences in the predicted probability of high DAS total scores for staff who have received all training compared to less training if they provide care in memory care units all of the time. However, assisted living staff who provide care none of the time in memory care units and completed informal training in all content areas had a higher predicted probability of DAS total compared to those who did not receive all training. Therefore, indicating that training may contribute to overall DAS especially for those not providing care in memory care unit in assisted living facilities.

**Table 4:** Logistic regressions of DAS and SCIDS with interactions of training by memory care unit.

Independent variables	DAS total		SCIDS total	
	Formal	Informal	Formal	Informal
	Nursing Home Staff (*N* = 355)			
Training	1.32 [0.22, 7.80]	0.83 [0.21, 3.23]	3.39 [1.34, 8.57]*	2.83 [0.79, 10.08]
Memory Care Unit				
All of the Time	0.79 [0.03, 23.49]	0.51 [0.03, 9.52]	0.89 [0.09, 9.00]	1.69 [0.14, 20.87]
Some of the Time	0.64 [0.08, 5.10]	0.76 [0.12, 4.71]	1.45 [0.24, 8.83]	0.75 [0.15, 3.71]
Training*All of the Time	0.99 [0.02, 45.10]	1.63 [0.07, 36.01]	1.09 [0.07, 16.48]	0.51 [0.04, 7.14]
Training*Some of the Time	2.37 [0.30, 18.66]	2.04 [0.28, 15.01]	1.25 [0.22, 6.97]	3.25 [0.43, 24.58]
	Assisted Living Staff (*N* = 404)			
Training	5.37 [0.65, 44.43]	5.14 [0.75, 35.03]	2.48 [0.23, 26.63]	2.64 [0.24, 28.87]
Memory Care Unit				
All of the Time	68.40 [4.18, 1,117.95]**	182.50 [30.05, 1,108.43]***	0.23 [0.01, 10.47]	3.86 [0.07, 220.22]
Some of the Time	0.45 [0.12, 1.74]	1.90 [0.23, 15.51]	0.22 [0.02, 2.45]	1.35 [0.09, 20.30]
Training*All of the Time	0.03 [0.0004, 1.30]	0.01 [0.001, 0.06]***	2.51 [0.04, 167.97]	0.12 [0.0007, 18.71]
Training*Some of the Time	1.58 [0.22, 11.31]	0.29 [0.02, 3.49]	3.24 [0.22, 48.18]	0.38 [0.01, 11.36]

Odds Ratios with 95% CI; weighted data; controlling for sociodemographic and work-related characteristics.

*** *p* < .001.

** *p* < .01.

* *p* < .05.

## 4 Discussion

Studies have documented that sufficient dementia care knowledge, positive attitudes, and appropriate practice are associated with patient and provider safety, improved symptom management, and high-quality care (6). In this brief report, we used national data from the U.S. to determine whether informal or formal training was associated with dementia care knowledge, attitudes, and practices for staff in nursing homes and assisted living facilities. The purpose of this study was to identify whether informal and formal training was related to dementia care competencies in order to assess how individual countries like the U.S. are meeting part of the actions outlined in the World Health Organization (2) action plan and where gaps remain.

### 4.1 Formal and informal training and dementia care outcomes

First, we found that staff working in both nursing homes and assisted living facilities reported high levels of training across all content areas (over 79% completed training in all content areas). However, the associations between training and dementia care outcomes differed across outcome domains and care settings. Specifically, formal training was associated with higher scores on both the DAS total and DAS person-centered knowledge subscale for staff in assisted living facilities. Formal training was also associated with higher scores on the DAS person-centered knowledge subscale for nursing home staff. These findings may reflect the nature of DAS, which captures dementia-related knowledge and person-centered attitudes that may be particularly responsive to structured or formal educational approaches (14). For example, formal training programs often emphasize person-centered philosophies of care, communication approaches, and conceptual understanding of dementia, which may strengthen workers' attitudes and beliefs about dementia care.

In contrast, among nursing home staff, both formal and informal training were associated with the SCIDS total and two subscales (i.e., professionalism and sustaining personhood). The SCIDS reflects workers' confidence in their dementia care practices, including maintaining professionalism and preserving personhood in care interactions. Informal learning experiences, including on-the-job learning, peer modeling, and accumulated experience, may contribute to workers' confidence in managing dementia care interactions and workplace responsibilities. Formal training may further reinforce these competencies by providing structured frameworks and approaches that workers can apply in care settings.

Notably, our results show that training was not significantly associated with SCIDS scores among staff at assisted living facilities, unlike the patterns observed in nursing homes possibly which may reflect the greater acuity and care complexity of nursing home residents relative to assisted living residents (18). The differences across long-term care settings may reflect the complexity of nursing home residents in amplifying the effects of training on workers' confidence in dementia care practices. In assisted living settings, where residents may have milder cognitive impairment and care demands are often less intensive, the competence-building effects of training may be weaker or less directly reflected in practice-oriented confidence measures compared to what we observed for dementia knowledge and attitudes.It is also possible that these association partly reflect underlying selection rather than training itself. Staff who participate in training may differ in motivation, or facility level resources/ investment in workforce development, factors not available in this dataset, and workers may self-select into settings based on preexisting attitudes toward dementia care. Given the cross-sectional design, unmeasured selection processes remain a possibly alternative explanation for these findings. We therefore encourage comparable interpretive caution for associations that align with our hypotheses as for those that do not.

### 4.2 Memory care unit context and dementia care comfort

The current study also identified important differences related to care provision in memory care units. Surprisingly, nursing home staff who provided care in memory care units all of the time are less likely to endorse the DAS comfort subscale items compared with those who provided care in memory care units none of the time. Because the DAS comfort items assess emotional and interpersonal comfort (e.g., feeling relaxed around persons living with dementia and finding dementia care rewarding), staff working in memory care units may experience greater emotional strain due to the demanding nature of advanced dementia care. Greater exposure to dementia care may therefore not necessarily be associated with higher emotional comfort, particularly in nursing home environments where residents often have more severe cognitive impairment and related behavioral symptoms (22).

In the interaction models examining whether memory care unit status moderates the association between different types of training and DAS scores, only informal training demonstrated a significant interaction effect. Specifically, assisted living staff who never worked in memory care units, but who had extensive informal training across all content areas had a higher predicted probability of high DAS scores compared to those who did not have informal training in all content areas. Among staff working in memory care units in assisted living facilities all of the time, DAS scores were relatively similar regardless of informal training exposure. These findings may suggest that informal learning opportunities are particularly important for staff with less routine exposure to specialized dementia care environments, and, therefore, an important area for investment.

### 4.3 Implications for dementia workforce training

The current findings have several implications for dementia workforce development. As the number of persons living with dementia continues to increase globally, identifying effective and accessible approaches to workforce training is a growing priority. The current findings suggest that formal and informal training may support different domains of dementia care competencies and that workforce development efforts may benefit from incorporating both structured educational content and experiential learning opportunities.

The findings also suggest that workplace context may shape how dementia training is experienced and reflected in workers' attitudes and confidence. Memory care units may require additional organizational support that address the emotional and interpersonal demands of advanced dementia care in addition to training alone. Recent work has also identified ongoing gaps in dementia workforce training standards, including limited attention to cultural safety, rural contexts, and healthcare worker self-care (23), highlighting the need for broader and more context-sensitive workforce development approaches.

### 4.4 Limitations

Due to the nature of secondary data, the current study was limited to measures available within the NDWS. The study was unable to assess important characteristics of training experiences, including frequency, duration, recency, quality, and modality of training. Second, because the data are cross-sectional and based on self-report, causal direction cannot be determined, including whether staff with more positive attitudes toward dementia care are more likely to work in certain care settings and complete training across all content areas.

## 5 Conclusion

In conclusion, formal and informal dementia training were differentially associated with dementia care knowledge, attitudes, and practice-related confidence across long-term care settings. Care context, particularly work in memory care units, may not necessarily translate into greater comfort in dementia care interactions without adequate training. Future research should replicate these findings across different countries and care contexts to provide more evidence for these associations especially for workforce outcomes over time.

## Data Availability

The NDWS includes both public use and retrstricted use data. Retstricted data was used for the current analysis. Information on data access is available here; https://www.ndws.org/.
